# Clinical characteristics of spontaneous intracranial basal ganglia hemorrhage and risk factors for hematoma expansion in the plateaus of China

**DOI:** 10.3389/fneur.2023.1183125

**Published:** 2023-06-15

**Authors:** Yujia Yan, Hecheng Ren, Bin Luo, Wanpeng Fan, Xiqiang Zhang, Ying Huang

**Affiliations:** ^1^Academy of Medical Engineering and Translational Medicine, Tianjin University, Tianjin, China; ^2^Department of Neurosurgery, Tianjin Huanhu Hospital, Tianjin, China; ^3^Department of Neurosurgery, Third People’s Hospital of Xining City, Xining, China

**Keywords:** plateau, intracranial cerebral hemorrhage, hematoma expansion, heterogeneity, risk factors

## Abstract

**Background and purpose:**

The clinical features of intracranial cerebral hemorrhage (ICH) and the risk factors for hematoma expansion (HE) have been extensively studied. However, few studies have been performed in patients who live on a plateau. The natural habituation and genetic adaptation have resulted in differences in disease characteristics. The purpose of this study was to investigate the differences and consistency of clinical and imaging characteristics of patients in the plateaus of China compared with the plains, and to analyze the risk factors for HE of intracranial hemorrhage in the plateau patients.

**Methods:**

From January 2020 to August 2022, we undertook a retrospective analysis of 479 patients with first-episode spontaneous intracranial basal ganglia hemorrhage in Tianjin and Xining City. The clinical and radiologic data during hospitalization were analyzed. Univariate and multivariate logistic regression analyzes were used to assess the risk factors for HE.

**Results:**

HE occurred in 31 plateau (36.0%) and 53 plain (24.2%) ICH patients, and HE was more likely to occur in the plateau patients compared with the plain (*p* = 0.037). The NCCT images of plateau patients also showed heterogeneity of hematoma imaging signs, and the incidence of blend signs (23.3% vs. 11.0%, *p* = 0.043) and black hole signs (24.4% vs. 13.2%, *p* = 0.018) was significantly higher than in the plain. Baseline hematoma volume, black hole sign, island sign, blend sign, and PLT and HB level were associated with HE in the plateau. Baseline hematoma volume and the heterogeneity of hematoma imaging signs were independent predictors of HE in both the plain and plateau.

**Conclusion:**

Compared with the plain, ICH patients in the plateau were more prone to HE. The patients showed the same heterogeneous signs on the NCCT images as in the plain, and also had predictive value for HE.

## Introduction

Hematoma expansion (HE) occurs in 40% of patients with untreated intracranial cerebral hemorrhage (ICH), and even a small amount of hematoma growth increases the chance of death and neurological deficits (1, 2). The clinical features of ICH and the risk factors associated with HE have been extensively studied, but most studies have been conducted in populations residing in plain cities.

The incidence of ICH in China was distributed in a north–south gradient, with a much higher incidence rate on the Qinghai-Tibetan plateau (3, 4). The highland resident population is different from the general population due to natural habituation, physiological compensation, and genetic adaptation to the unique environment (low oxygen, low atmospheric pressure, low temperature, and intense ultraviolet radiation, etc.) over a long period of time, which makes them unique (5).

There is a lack of description of ICH in the highlands. Are the existing risk factors for predicting HE and the heterogeneity of hematoma imaging signs in non-contrast computed tomography (NCCT) also applicable to the plateau? The answers to such questions have not been reported in studies. The purpose of this study was to investigate the consistency and differences of clinical and imaging features of ICH in the Chinese plateaus and plains, and analyze the risk factors for HE in the plateaus, to provide a reference for treatment decisions for patients with ICH in the plateau.

## Materials and methods

### Study design and participants

We retrospectively collected demographic, clinical, laboratory, and radiographic data of 479 patients with spontaneous ICH in the basal ganglia region admitted in Tianjin and Xining City from January 2021 to August 2022. Baseline NCCT scan and laboratory data were collected 6–12 h after the onset; a follow-up NCCT scan was performed within 24–48 h after onset. The diagnostic criteria were in accordance with the Guidelines for the Management of Spontaneous Intracerebral Hemorrhage (6).

Inclusion criteria were as follows (1): age ≥ 18, <85 years old (2); patients with first-onset spontaneous ICH; and (3) baseline NCCT scan within 6–12 h of the onset of ICH symptoms, and the follow-up NCCT scan performed within 24–48 h after the onset. Exclusion criteria were (1): patients who were not local residents, and with a lack of available data (2); patients who underwent surgery treatment before the follow-up NCCT scan (3); patients with ICH secondary to head trauma, arteriovenous malformation, cerebral aneurysm, brain tumor, venous sinus embolism, or ischemic stroke (4); intraventricular hemorrhage (IVH) (5); concomitant subarachnoid hemorrhages (6); hematoma volume < 1 mL; or (7) patients who took long-term antithrombotic medications, such as anticoagulation and/or antiplatelet therapy.

**Figure 1:**
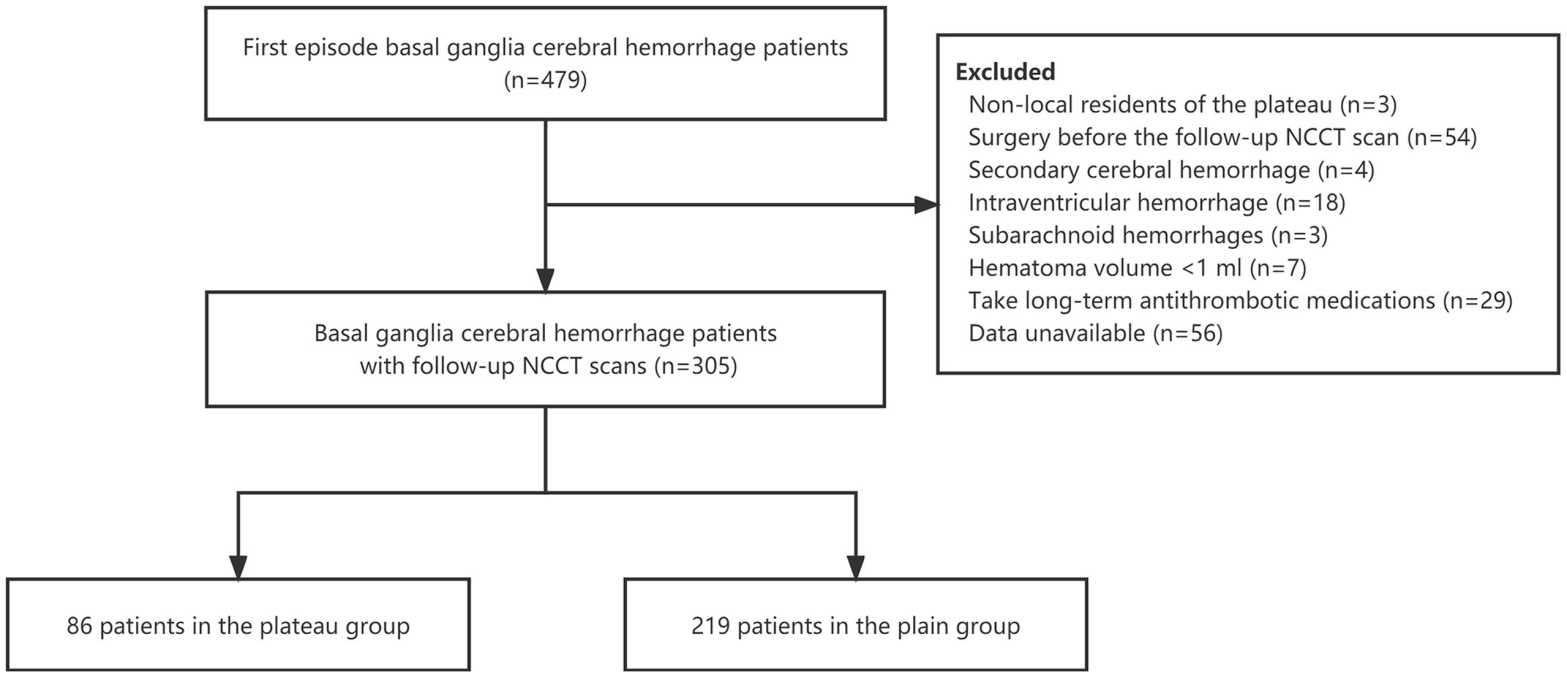
Flowchart of participants’ selection.

Overall, 305 patients were included in the study (Figure 1). In the plateau group were 86 patients from the Third People Hospital of Xining City, Qinghai Province (average altitude 2261.2 m); 219 patients from the Tianjin Huanhu Hospital (average altitude 3.3 m) were the plain group. The study was approved by the ethics committees of Third People Hospital of Xining City and Tianjin Huanhu Hospital, and did not require written informed consent.

### Imaging process and assessment

**Figure 2:**
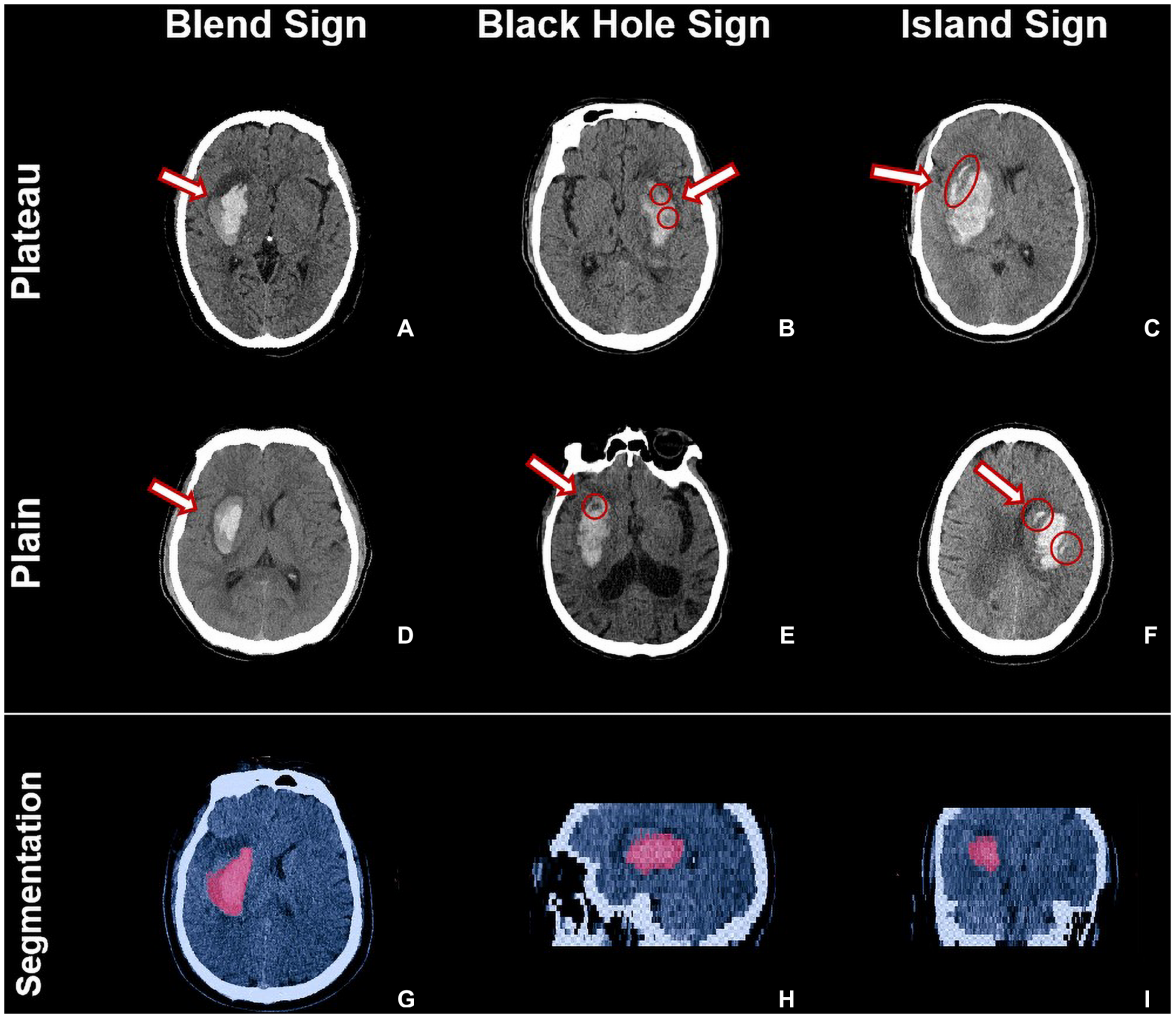
The illustration of heterogeneous signs and the segmentation of hematoma lesions through ITK-SNAP software. The red arrows and circles in **A–C**, and **D–F** marked the blend sign, black hole sign, and island sign of hemorrhage in the plateau and plain, respectively. **G–I** images are shown segmented from the cross-sectional, sagittal, and coronal planes of the hematoma lesions, respectively.

Head NCCT images were processed using ITK-SNAP software.^1^ We used semi-automatic and manual forms to label hematoma lesions in cross-sectional, coronal, and sagittal planes, respectively, and to perform volume calculations and 3D image imaging renderings (7). HE was defined as a 6 mL increase in hematoma volume or a 33% increase in hematoma volume from the onset (8). The heterogeneity of hematoma and image markers such as island sign, blend sign, and black hole sign was determined according to the criteria of Li et al. (9–11) (Figure 2).

### Statistical analysis

All statistical analyzes were performed by using a commercially available software package (SPSS Version 25.0). Shapiro–Wilk test was used to assess the normality. Continuous variables were reported as mean with SD or median (interquartile ranges, IQR), and categorical variables were reported as frequencies (percentages). We used independent *t* tests or the Mann–Whitney *U* tests for continuous variables, and Pearson χ2 or Fisher exact tests for categorical variables to compare the groups. Spearman’s rank correlation analysis was used for correlation analysis. Independent association of the variables and hematoma expansion was evaluated by using univariate logistic regression and multivariable logistic regression. *p* < 0.05 was considered statistically significant. Kappa test was used to calculate κ values for assessing the interobserver reliability of the heterogeneity of hematoma imaging signs. *κ* = 1 indicates total agreement between observers.

## Results

### Baseline demographic and clinical characteristics

A total of 305 patients with spontaneous intracranial basal ganglia hemorrhage were integrated into our study. To clarify the selection bias, baseline characteristics between included and excluded patients are shown in Supplemental Table S1.

There were no differences in demographic characteristics (age, sex, and ethnicity distribution) between the plateau and plain patients. The plateau patients had a significantly higher prevalence of hypertension (diastolic blood pressure), hyperlipidemia (TC, TG, LDL-C), erythrocytosis (RBC, HB), and alcohol abuse, and lower oxygen saturation level and diabetes. In coagulation function, FIB levels were significantly decreased, and PT and TT were prolonged in patients from high altitudes. Moreover, in the plateau patients, the baseline hematoma volume was significantly larger (Median, 16.4 vs. 12.2, *p* = 0.011) and there were more severe neurological impairments (Median, GCS 11 vs. 13, *p* < 0.001; Table 1).

**Table 1:** Baseline demographic and clinical characteristics between the plain and plateau patients.

	Plain (*n* = 219)	Plateau (*n* = 86)	*P* Value
Age, y (SD)	57.3 (14.9)	55.5 (11.6)	0.270
Male sex (%)	146 (66.7)	53 (61.6)	0.406
Race (%)			0.376
Han	210 (95.9)	80 (93.0)	
Other	9 (4.1)	6 (7.0)	
Current Smoking (%)	98 (44.7)	35 (40.7)	0.521
Current Drinking (%)	77 (35.2)	53 (61.6)	**<0.001** *
Vital signs			
Respiratory rate (times/min)	17 (3)	18 (2)	0.129
Heart rate (beats/min)	77 (23)	76 (20)	0.784
Oxyhemoglobin saturation (%)	98 (3)	96 (2)	**<0.001** *
Hypertension (%)	139 (63.5)	66 (76.7)	**0.026** *
Systolic blood pressure (mmHg)	159 (30)	161 (33)	0.315
Diastolic blood pressure (mmHg)	95 (15)	100 (17)	**<0.001** *
Diabetes mellitus (%)	95 (43.4)	24 (27.9)	**0.013** *
Glucose level (mmol/L)	6.8 (2.5)	6.4 (1.8)	**<0.001** *
Hyperlipidemia (%)	97 (44.3)	58 (67.4)	**<0.001** *
TC (mmol/L)	5.2 (1.1)	5.4 (1.0)	**0.012** *
TG (mmol/L)	1.3 (0.9)	1.8 (1.9)	**<0.001** *
LDL-C (mmol/L)	3.0 (1.1)	3.3 (1.1)	**0.024** *
HDL-C (mmol/L)	1.3 (0.5)	1.1 (0.3)	**0.005** *
Coronary artery disease (CHD) (%)	79 (36.1)	37 (43.0)	0.261
Erythrocytosis (%)	2 (0.9)	13 (15.1)	**<0.001** *
RBC (*10^12^/L)	4.5 (0.9)	4.9 (0.9)	**<0.001** *
HB (g/L)	146 (28)	166 (29)	**<0.001** *
PLT(*10^9^/L)	206 (80)	163 (63)	**<0.001** *
HCT/pcv (%)	0.4 (0.1)	0.5 (0.1)	**<0.001** *
Blood coagulation			
APTT (s)	23.7 (4.2)	23.4 (3.4)	0.115
PT (s)	11.8 (2.1)	12.8 (1.5)	**<0.001** *
FIB (g/L)	2.8 (1.0)	2.3 (1.2)	**<0.001** *
TT (s)	16.9 (1.6)	17.4 (1.9)	**0.032** *
INR	1.0 (0.2)	1.0 (0.2)	0.232
Baseline hematoma volume (ml)	12.2 (14.1)	16.4 (14.8)	**0.011** *
GCS	13 (2)	11 (3)	**<0.001** *
Hematoma expansion (HE) (%)	53 (24.2)	31 (36.0)	**0.037** *
Black hole sign (%)	29 (13.2)	21 (24.4)	**0.018** *
Island sign (%)	14 (6.4)	8 (9.3)	0.377
Blend sign (%)	24 (11.0)	20 (23.3)	**0.043** *

Data expressed as mean (SD), median (IQR) or n (%).

* *p* < 0.05 was considered statistically significant. The bold values was considered statistically significant.

### Non-contrast computed tomography imaging characteristics

HE occurred in 31 patients with ICH in the plateau group (36.0, 95%CI 25.7–46.4%) and 53 patients in the plain group (24.2, 95%CI 18.5–29.9%). HE was more likely to occur in the plateau patients compared with the plain (*p* = 0.037). In the plateau group, 20 patients with blend sign (*к* = 0.872), 21 patients with black hole sign (*к* = 0.897), and eight patients with island sign (*к* = 0.864) were observed; in the plain group, 24 with blend sign (*к* = 0.851), 29 with black hole sign (*к* = 0.880), and 14 with island sign (*к* = 0.894) were observed. The NCCT images of patients with ICH in the plateau also showed heterogeneity of hematoma imaging signs, and the incidence of blend sign (23.3% vs. 11.0%, *p* = 0.043) and black hole sign (24.4% vs. 13.2%, *p* = 0.018) was significantly higher than in the plain patients.

### Predictors of hematoma expansion in the plain and plateau

Univariate analysis showed that the systolic blood pressure, baseline hematoma volume, GCS, black hole sign, island sign, and blend sign were associated with HE in the plain patients; diastolic blood pressure, HB, PLT, baseline hematoma volume, GCS, black hole sign, island sign, and blend sign were associated with HE in the plateau patients. Multivariate analysis showed that, differently from plain patients, PLT and HB level were independent risk factors for HE in the plateau; baseline hematoma volume and the heterogeneity of hematoma imaging signs (black hole sign, island sign, and blend sign) were independent predictors of HE in both the plain and plateau patients (Table 2).

**Table 2:** Univariate and multivariate analysis of predictors for hematoma expansion in the plain and plateau patients.

	Plain		Univariate analysis	Multivariate analysis	OR (95% CI)	Plateau		Univariate analysis	Multivariate analysis	OR (95% CI)
	Non-HE (*n* = 166)	HE (*n* = 53)				Non-HE (*n* = 55)	HE (*n* = 31)			
Age, y (SD)	57.3 (14.8)	57.4 (15.4)	0.960			55.6 (11.6)	55.3 (11.9)	0.896		
Male sex (%)	108 (65.1)	38 (66.0)	0.372			31 (56.4)	22 (71.0)	0.181		
Current Smoking (%)	74 (44.6)	24 (45.3)	0.928			24 (43.6)	11 (35.5)	0.460		
Current Drinking (%)	58 (34.9)	19 (35.8)	0.904			33 (60.0)	20 (64.5)	0.679		
Hypertension (%)	107 (64.5)	32 (60.4)	0.591			41 (74.5)	25 (80.6)	0.520		
Systolic blood pressure (mmHg)	156 (27)	168 (34)	**0.021** *	0.414		157 (27)	166 (38)	0.102		
Diastolic blood pressure (mmHg)	94 (16)	97 (13)	0.104			99 (15)	100 (20)	**0.043** *	0.510	
Diabetes mellitus (%)	72 (43.4)	23 (43.4)	0.998			15 (27.3)	9 (29.0)	0.861		
Glucose level (mmol/L)	6.8 (2.2)	6.9 (3.7)	0.166			6.1 (1.7)	6.4 (1.9)	0.719		
Hyperlipidemia (%)	72 (43.4)	25 (47.2)	0.628			37 (67.3)	21(67.7)	0.964		
TC (mmol/L)	5.1 (1.2)	5.3 (0.9)	0.647			5.4 (1.0)	5.5 (1.0)	0.770		
TG (mmol/L)	1.3 (0.9)	1.3 (0.9)	0.557			1.7 (1.5)	2.2 (2.6)	0.088		
LDL-C (mmol/L)	3.0 (1.2)	2.8 (1.0)	0.479			3.4 (1.2)	3.1 (1.2)	0.442		
HDL-C (mmol/L)	1.2 (0.4)	1.4 (0.6)	0.438			1.1 (0.3)	1.2 (0.2)	0.494		
Coronary artery disease (CHD) (%)	54 (32.5)	25 (47.2)	0.053			20 (36.4)	17 (54.8)	0.097		
Erythrocytosis (%)	2 (1.2)	0	>0.999			9 (16.4)	4 (12.9)	0.667		
RBC (*10^12^/L)	4.5 (0.9)	4.4 (1.0)	0.159			4.9 (1.3)	4.9 (0.5)	0.388		
HB (g/L)	141 (28)	152 (24)	0.191			158 (36)	173 (14)	**0.010** *	**0.005** *	1.07 (1.02–1.22)
PLT(*10^9^/L)	206 (84)	200 (82)	0.672			173 (84)	149 (25)	**0.002** *	**0.038** *	0.98 (0.96–0.99)
HCT/pcv (%)	0.4 (0.1)	0.4 (0.1)	0.845			0.5 (0.1)	0.5 (0.1)	0.514		
Blood coagulation										
APTT (s)	23.6 (4.4)	23.9 (3.1)	0.220			23.8 (3.6)	22.6 (3.3)	0.299		
PT (s)	11.8 (2.0)	11.8 (2.4)	0.891			13.4 (1.6)	12.7 (1.3)	0.258		
FIB (g/L)	2.8 (1.0)	2.8 (1.1)	0.661			2.3 (1.5)	2.4 (1.0)	0.808		
TT (s)	16.9 (1.5)	17.0 (1.6)	0.753			17.3 (1.7)	17.9 (2.4)	0.272		
INR	1.0 (0.2)	1.0 (0.3)	0.986			1.0 (0.2)	1.0 (0.3)	0.439		
Baseline hematoma volume (ml)	10.1 (10.0)	22.7 (7.1)	**<0.001** *	**0.018** *	1.15 (1.02–1.28)	14.5 (14.3)	22.1 (13.3)	**0.011** *	**0.021** *	1.07 (1.01–1.12)
GCS	13 (2)	11 (3)	**<0.001** *	0.810		12 (3)	11(4)	**0.011** *	0.363	
Black hole sign (%)	10 (6.0)	19 (35.8)	**<0.001** *	**0.030** *	3.28 (1.12–9.62)	7 (12.7)	14 (45.2)	**0.001** *	**0.004** *	9.87 (2.06–47.26)
Island sign (%)	2 (1.2)	12 (22.6)	**<0.001** *	**0.001** *	17.33 (3.13–95.78)	2 (3.6)	6 (19.4)	**0.016** *	**0.010** *	27.19 (2.21–334.60)
Blend sign (%)	9 (5.4)	15 (28.3)	**<0.001** *	**0.019** *	4.03 (1.26–12.85)	7 (12.7)	13 (41.9)	**0.002** *	**0.011** *	7.63 (1.60–36.30)

Data expressed as mean (SD), median (IQR), or *n* (%).

CI indicates confidence interval; OR, odds ratio; HE, hematoma expansion.

* *p* < 0.05 was considered statistically significant. The bold values was considered statistically significant.

## Discussion

In the present study, we found differences and consistencies in the clinical and imaging features of hemorrhage in the basal ganglia region of the plateau compared with the plain patients. Patients from the plateau were more prone to HE than the plain, showing the same heterogeneity of hematoma imaging signs on the NCCT images. Baseline hematoma volume and the heterogeneity of hematoma imaging signs were independent risk factors for HE in spontaneous intracranial basal ganglia hemorrhage in both the plain and plateau patients.

In the plateau, the cold weather and traditional diet, characterized by high fat, high salt, and large intake of strong liquor, greatly increase the risk of cerebrovascular accidents and poor prognosis (12). Baseline clinical characteristics of patients in the plateau were significantly different compared to the plain patients, with a higher prevalence of hypertension, hyperlipidemia, erythrocytosis, and alcohol abuse, and lower diabetes. The epidemiological and metabolic characteristics of patients in the plateau have been reported in detail in our previous studies (5). The incidence of ICH is higher in the plateau of China, especially the Qinghai-Tibet Plateau, compared to the more medically and economically developed southeastern China. Lack of economic development, uneven medical resources, and a long pre-hospital rescue radius make early identification of risk factors for HE important for making therapeutic strategies and improving the outcome of ICH in the plateau (13).

Baseline hematoma volume is one of the risk factors for predicting HE, as well as a key predictor of patient mortality and prognosis (14, 15). In the present study, the findings were consistent with current reports that baseline hematoma volume was one of the independent risk factors for HE in the plateau ICH population, and was significantly higher than in plains. The baseline hematoma volume is mainly influenced by the level of arterial blood pressure (6, 16). In the relationship between arterial blood pressure and cerebrovascular accidents, diastolic blood pressure is thought to independently influence the risk of cardiovascular events and adverse outcomes (17). The plateau patients in this study exhibited significant high diastolic blood pressure (Median, 100 vs. 95, *p* < 0.001), which may be related to the elevated erythrocyte and hemoglobin counts compensated by hypoxia and increased blood viscosity leading to increased peripheral resistance. Therefore, in the prevention of cerebral hemorrhage and daily management of blood pressure in plateau areas, it is recommended that people living in plateau regions improve their dietary structure, reduce their salt and fat intake, and pay attention to the monitoring and management of blood pressure, especially diastolic blood pressure, which will have a positive effect on reducing the occurrence of cerebral hemorrhage disease in high altitude areas.

In addition, the decreased PT and PLT may lead to a state of increased bleeding and inconsistent hematoma density in patients, which increases the risk of re-bleeding in patients with cerebral hemorrhage by 2–3 times (18). Activated platelets involved in adhesion, aggregation, release, and coagulation of thrombus formation during cerebral hemorrhage are heavily depleted, resulting in a significant decrease in peripheral platelets (19). In this study, we found that the PT and PLT count were lower in patients with cerebral hemorrhage in the plateau, which suggests that we need to pay attention to the coagulation function of patients and adjust the dose of prothrombin used by patients in a timely manner to avoid HE due to excessive platelet consumption and abnormal coagulation function.

The density of hematoma is influenced by their composition, where the hemoglobin content is an important determinant of hematoma density on NCCT images, which shows high density when the blood is clotted and lower density when there is active bleeding, while the mixing of blood at different times leads to the appearance of heterogeneous signs of high and low mixed density (9, 10). Heterogeneous signs were positively associated with early HE, poor prognosis, morbidity, and mortality (20). Meta-analysis showed that the black hole sign had a 30% sensitivity and 91% specificity in predicting HE; the blend sign had a sensitivity of 28% and a specificity of 92% in making predictions (21, 22). However, the results of the current study are from patients from the plains. Patients residing on the plateau have a compensatory increase in erythrocyte and hemoglobin content to increase the oxygen-carrying capacity of the blood and the oxygen-supplying capacity of the tissues due to the increased bone marrow hematopoiesis stimulated by long-term chronic hypoxia (23). Whether the same heterogeneous signs manifest on NCCT images in patients with ICH in the plateau and whether they are predictive of HE have not been reported.

In this study, we analyzed the heterogeneous signs in NCCT images of patients with ICH at different altitudes. In the plateau, the incidence of blend sign (23.3% vs. 11.0%) and black hole sign (24.4% vs. 13.2%) was significantly higher than that in the plain patients. In the prediction of HE, the heterogeneous sign was an independent risk factor. Similar to the results of the current study based on plain patients, it indicates that the heterogeneous signs summarized by imaging of plain cerebral hemorrhage patients are also applicable to the plateau and have predictive value for HE.

In this study, there are several limitations to consider. We investigated the differences in clinical and imaging characteristics of patients in the plateaus of China compared with the plains. With the inherent defects of retrospective analysis and sample size, we need to perform a larger multi-center study to validate the results. In addition, this study only included brain hemorrhage in the basal ganglia region, so the findings can only be interpreted cautiously within this context. Due to the limitations of the medical status and facilities in the highlands, this study only illustrates the clinical manifestations. Therefore, we need to perform a more molecular description of the condition after bleeding in both populations and analyze the differences of the mechanisms of HE between the plateau and plain patients.

## Conclusion

In this study we found that patients with untreated ICH in the basal ganglia region on the plateau were more prone to HE. Compared with the plain, ICH in the plateau shows specificity in the clinical features and consistency in the predictive value of the heterogeneous signs of NCCT. This study provides validation of the generalizability and application value of current clinical studies to predict HE based on patients from the plain. Therefore, in the plateau ICH patients, we can assess early HE using heterogeneous signs. In patients with blend, black hole, and/or island signs, we suggest aggressive treatment to help prevent further neurological deterioration.

## Data availability statement

The original contributions presented in the study are included in the article/Supplementary material, further inquiries can be directed to the corresponding author.

## Ethics statement

The studies involving human participants were reviewed and approved by The Ethics Committees of Third People Hospital of Xining City and Tianjin Huanhu Hospital. Written informed consent for participation was not required for this study in accordance with the national legislation and the institutional requirements.

## Author contributions

YY, BL, and YH: study concept and design. YY and BL: data acquisition. YY, BL, and WF: statistical analysis. WF and XZ: interpretation of data. YY and BL: manuscript drafting. YY and YH: review and editing. YY and HCR: revised. YH: supervision. All authors contributed to the article and approved the submitted version.

## Funding

This work was supported by the Xining Science and Technology Program of China (Grant No. 2022-M-35).

## Conflict of interest

The authors declare that the research was conducted in the absence of any commercial or financial relationships that could be construed as a potential conflict of interest.

## Publisher’s note

All claims expressed in this article are solely those of the authors and do not necessarily represent those of their affiliated organizations, or those of the publisher, the editors and the reviewers. Any product that may be evaluated in this article, or claim that may be made by its manufacturer, is not guaranteed or endorsed by the publisher.

## Footnotes

^1^ http://www.itksnap.org

## References

[1] Samarasekera N Fonville A Lerpiniere C Farrall AJ Wardlaw JM White PM. Influence of intracerebral hemorrhage location on incidence, characteristics, and outcome: population-based study. Stroke. (2015) 46:361–8. doi: 10.1161/STROKEAHA.114.007953, PMID: 25586833

[2] Van Asch CJ Luitse MJ Rinkel GJ van der Tweel I Algra A Klijn CJ. Incidence, case fatality, and functional outcome of intracerebral haemorrhage over time, according to age, sex, and ethnic origin: a systematic review and meta-analysis. Lancet Neurol. (2010) 9:167–76. doi: 10.1016/S1474-4422(09)70340-0, PMID: 20056489

[3] Zhang S Liu D Gesang DZ Lv M. Characteristics of cerebral stroke in the Tibet autonomous region of China. Med Sci Monit. (2020) 26:e919221. doi: 10.12659/MSM.919221, PMID: 31917778 PMC6977622

[4] Xu G Ma M Liu X Hankey GJ. Is there a stroke belt in China and why? Stroke. (2013) 44:1775–83. doi: 10.1161/strokeaha.113.001238 23674531

[5] Yan Y Zhang X Ren H An X Fan W Liang J. Anterior circulation acute ischemic stroke in the plateau of China: risk factors and clinical characteristics [original research]. Front Neurol. (2022) 13:13. doi: 10.3389/fneur.2022.859616 PMC9043326 35493834

[6] Hemphill JC 3rd Greenberg SM Anderson CS Becker K Bendok BR Cushman M. Guidelines for the Management of Spontaneous Intracerebral Hemorrhage: a guideline for healthcare professionals from the American Heart Association/American Stroke Association. Stroke. (2015) 46:2032–60. doi: 10.1161/STR.0000000000000069, PMID: 26022637

[7] Yushkevich PA Piven J Hazlett HC Smith RG Ho S Gee JC. User-guided 3D active contour segmentation of anatomical structures: significantly improved efficiency and reliability. NeuroImage. (2006) 31:1116–28. doi: 10.1016/j.neuroimage.2006.01.015, PMID: 16545965

[8] Dowlatshahi D Demchuk AM Flaherty ML Ali M Lyden PL Smith EE. Defining hematoma expansion in intracerebral hemorrhage: relationship with patient outcomes. Neurology. (2011) 76:1238–44. doi: 10.1212/WNL.0b013e3182143317, PMID: 21346218 PMC3068004

[9] Li Q Zhang G Huang YJ Dong MX Lv FJ Wei X. Blend sign on computed tomography: novel and reliable predictor for early hematoma growth in patients with intracerebral hemorrhage. Stroke. (2015) 46:2119–23. doi: 10.1161/STROKEAHA.115.009185, PMID: 26089330

[10] Li Q Zhang G Xiong X Wang XC Yang WS Li KW. Black hole sign: novel imaging marker that predicts hematoma growth in patients with intracerebral hemorrhage. Stroke. (2016) 47:1777–81. doi: 10.1161/STROKEAHA.116.013186, PMID: 27174523

[11] Li Q Liu QJ Yang WS Wang XC Zhao LB Xiong X. Island sign: An imaging predictor for early hematoma expansion and poor outcome in patients with intracerebral hemorrhage. Stroke. (2017) 48:3019–25. doi: 10.1161/STROKEAHA.117.017985, PMID: 29018128

[12] Zhao Y Yao Z D'Souza W Zhu C Chun H Zhuoga C. An epidemiological survey of stroke in Lhasa, Tibet. China Stroke. (2010) 41:2739–43. doi: 10.1161/strokeaha.110.586669, PMID: 20966420

[13] Du CN Yang MF Zhang Q Jin XQ Yan C Huang YW. Establishment and verification of the hematoma expansion prediction score of intracerebral hemorrhage in the Qinghai-Tibetan plateau. World Neurosurg. (2019) 123:e465–73. doi: 10.1016/j.wneu.2018.11.189, PMID: 30500588

[14] Al-Shahi Salman R Law ZK Bath PM Steiner T Sprigg N. Haemostatic therapies for acute spontaneous intracerebral haemorrhage. Cochrane Database Syst Rev. (2018) 4:Cd005951. doi: 10.1002/14651858.CD005951.pub4 29664991 PMC6494564

[15] Hillal A Ullberg T Ramgren B Wassélius J. Computed tomography in acute intracerebral hemorrhage: neuroimaging predictors of hematoma expansion and outcome. Insights. Imaging. (2022) 13:180. doi: 10.1186/s13244-022-01309-1, PMID: 36417131 PMC9684397

[16] Li W Jin C Vaidya A Wu Y Rexrode K Zheng X. Blood pressure trajectories and the risk of intracerebral hemorrhage and cerebral infarction: a prospective study. Hypertension. (2017) 70:508–14. doi: 10.1161/HYPERTENSIONAHA.117.09479, PMID: 28716992 PMC5720155

[17] Whelton PK Carey RM Aronow WS Casey DE Collins KJ Dennison Himmelfarb C. ACC/AHA/AAPA/ABC/ACPM/AGS/APhA/ASH/ASPC/NMA/PCNA guideline for the prevention, detection, evaluation, and Management of High Blood Pressure in adults: a report of the American College of Cardiology/American Heart Association task force on clinical practice guidelines. Hypertension. (2017) 71:e13–e115. doi: 10.1161/hyp.0000000000000065 29133356

[18] Kuramatsu JB Sembill JA Huttner HB. Reversal of oral anticoagulation in patients with acute intracerebral hemorrhage. Crit Care. (2019) 23:206. doi: 10.1186/s13054-019-2492-8, PMID: 31171018 PMC6555738

[19] Ziai WC Torbey MT Kickler TS Oh S Bhardwaj A Wityk RJ. Platelet count and function in spontaneous intracerebral hemorrhage. J Stroke Cerebrovasc Dis. (2003) 12:201–6. doi: 10.1016/S1052-3057(03)00075-2, PMID: 17903927

[20] Li R Yang M. A comparative study of the blend sign and the black hole sign on CT as a predictor of hematoma expansion in spontaneous intracerebral hemorrhage. Biosci Trends. (2017) 11:682–7. doi: 10.5582/bst.2017.01283, PMID: 29311451

[21] Zheng J Yu Z Guo R Li H You C Ma L. Meta-analysis of predictive significance of the black hole sign for hematoma expansion in intracerebral hemorrhage. World Neurosurg. (2018) 115:e711–6. doi: 10.1016/j.wneu.2018.04.140, PMID: 29709738

[22] Yu Z Zheng J Guo R Ma L Li M Wang X. Performance of blend sign in predicting hematoma expansion in intracerebral hemorrhage: a meta-analysis. Clin Neurol Neurosurg. (2017) 163:84–9. doi: 10.1016/j.clineuro.2017.10.017, PMID: 29078128

[23] Wang W Jiang B Sun H Ru X Sun D Wang L. Prevalence, incidence, and mortality of stroke in China: results from a Nationwide population-based survey of 480 687 adults. Circulation. (2017) 135:759–71. doi: 10.1161/CIRCULATIONAHA.116.025250, PMID: 28052979

