# Supplementary material for: Clinical characteristics of spontaneous intracranial basal ganglia hemorrhage and risk factors for hematoma expansion in the plateaus of China
Source: Front Neurol. 2023 Jun 15;14:1183125. doi: 10.3389/fneur.2023.1183125 (PMC10313382; doi:10.3389/fneur.2023.1183125)
Supplement: Supplementary file 1 [file Table_1.docx]

**Supplemental Table 1** The baseline characteristics between the included and excluded patients.

|  | | **Included (n=305)** | **Excluded (n=174)** | ***P* Value** |
| --- | --- | --- | --- | --- |
| Age, y (SD) | | 56.8 (14.0) | 57.5 (12.7) | 0.596 |
| Male sex (%) | | 199 (65.2) | 115 (66.1) | 0.851 |
| Race (Han) (%) | | 290 (95.1) | 169 (97.1) | 0.282 |
| Current Smoking (%) | | 133 (43.6) | 85 (48.9) | 0.268 |
| Current Drinking (%) | | 130 (42.6) | 69 (39.7) | 0.526 |
| Vital signs | |  |  |  |
|  | Respiratory rate (times/minute) | 17 (3) | 18 (3) | 0.859 |
|  | Heart rate (beats/minute) | 76 (22) | 76 (21) | 0.952 |
|  | Oxyhemoglobin saturation (%) | 97 (3) | 97 (3) | 0.115 |
| Hypertension (%) | | 205 (67.2) | 125 (71.8) | 0.293 |
|  | Systolic blood pressure (mmHg) | 160 (31) | 160 (27) | 0.641 |
|  | Diastolic blood pressure (mmHg) | 95 (16) | 95 (16) | 0.806 |
| Diabetes mellitus (%) | | 119 (39.1) | 66 (37.9) | 0.814 |
|  | Glucose level (mmol/L) | 6.7 (2.3) | 6.7 (1.7) | 0.802 |
| Hyperlipidemia (%) | | 155 (50.8) | 89 (51.1) | 0.945 |
|  | TC (mmol/L) | 5.3 (1.1) | 5.3 (1.2) | 0.791 |
|  | TG (mmol/L) | 1.3 (1.0) | 1.3 (1.1) | 0.560 |
|  | LDL-C (mmol/L) | 3.0 (1.2) | 3.1 (1.2) | 0.288 |
|  | HDL-C (mmol/L) | 1.2 (0.4) | 1.2 (0.4) | 0.218 |
| Coronary artery disease (CHD) (%) | | 116 (38.0) | 59 (33.9) | 0.367 |
| Erythrocytosis (%) | | 15 (4.9) | 6 (3.4) | 0.450 |
|  | RBC (*10^12^/L) | 4.6 (1.0) | 4.5 (0.7) | 0.344 |
|  | HB (g/L) | 151 (33) | 149 (28) | 0.145 |
|  | PLT(*10^9^/L) | 198 (85) | 198 (64) | 0.495 |
|  | HCT/pcv (%) | 0.5 (0.1) | 0.4 (0.1) | 0.066 |
| Blood coagulation | |  |  |  |
|  | APTT (s) | 23.6 (3.9) | 23.6 (4.0) | 0.982 |
|  | PT (s) | 12.3 (2.1) | 12.1 (3.0) | 0.892 |
|  | FIB (g/L) | 2.7 (1.1) | 2.6 (1.0) | 0.337 |
|  | TT (s) | 17.1 (1.7) | 16.9 (1.4) | 0.270 |
|  | INR | 1.0 (0.2) | 1.0 (0.1) | 0.207 |
| Baseline hematoma volume (ml) | | 13.1 (14.9) | 11.5 (14.7) | 0.188 |
| GCS | | 13 (3) | 13 (3) | 0.869 |

Data expressed as mean (SD), median (IQR) or n (%).

**P*< 0.05 was considered statistically significant.
